# Role of Myostatin in Muscle Degeneration by Random Positioning Machine Exposure: An *in vitro* Study for the Treatment of Sarcopenia

**DOI:** 10.3389/fphys.2022.782000

**Published:** 2022-02-03

**Authors:** Ida Cariati, Manuel Scimeca, Roberto Bonanni, Rebecca Triolo, Valerio Naldi, Giuseppe Toro, Mario Marini, Virginia Tancredi, Riccardo Iundusi, Elena Gasbarra, Umberto Tarantino

**Affiliations:** ^1^Ph.D. in Medical-Surgical Biotechnologies and Translational Medicine, “Tor Vergata” University of Rome, Rome, Italy; ^2^Department of Clinical Sciences and Translational Medicine, “Tor Vergata” University of Rome, Rome, Italy; ^3^Department of Biomedicine and Prevention, “Tor Vergata” University of Rome, Rome, Italy; ^4^Department of Orthopaedics and Traumatology, “Policlinico Tor Vergata” Foundation, Rome, Italy; ^5^Department of Medical and Surgical Specialties and Dentistry, University of Campania “Luigi Vanvitelli”, Naples, Italy; ^6^Department of Systems Medicine, “Tor Vergata” University of Rome, Rome, Italy; ^7^Centre of Space Bio-Medicine, “Tor Vergata” University of Rome, Rome, Italy

**Keywords:** satellite cells, myostatin, muscle degeneration, sarcopenia, random positioning machine

## Abstract

Several scientific evidence have shown that exposure to microgravity has a significant impact on the health of the musculoskeletal system by altering the expression of proteins and molecules involved in bone–muscle crosstalk, which is also observed in the research of microgravity effect simulation. Among these, the expression pattern of myostatin appears to play a key role in both load-free muscle damage and the progression of age-related musculoskeletal disorders, such as osteoporosis and sarcopenia. Based on this evidence, we here investigated the efficacy of treatment with anti-myostatin (anti-MSTN) antibodies on primary cultures of human satellite cells exposed to 72 h of random positioning machine (RPM). Cell cultures were obtained from muscle biopsies taken from a total of 30 patients (controls, osteoarthritic, and osteoporotic) during hip arthroplasty. The Pax7 expression by immunofluorescence was carried out for the characterization of satellite cells. We then performed morphological evaluation by light microscopy and immunocytochemical analysis to assess myostatin expression. Our results showed that prolonged RPM exposure not only caused satellite cell death, but also induced changes in myostatin expression levels with group-dependent variations. Surprisingly, we observed that the use of anti-MSTN antibodies induced a significant increase in cell survival after RPM exposure under all experimental conditions. Noteworthy, we found that the negative effect of RPM exposure was counteracted by treatment with anti-MSTN antibodies, which allowed the formation of numerous myotubes. Our results highlight the role of myostatin as a major effector of the cellular degeneration observed with RPM exposure, suggesting it as a potential therapeutic target to slow the muscle mass loss that occurs in the absence of loading.

## Introduction

Sarcopenia is a typical pathological condition of aging characterized by a progressive reduction in muscle strength and function leading to physical frailty and an increased risk of falls and fractures (Cariati et al., 2021). The molecular mechanisms underlying the development and progression of sarcopenia include nuclear apoptosis, mitochondrial dysfunction, and muscle fiber denervation (Deschenes, 2004; Alway and Siu, 2008; Marzetti et al., 2013). Noteworthy, mitochondrial DNA damage in a mouse model has been reported to result in loss of the satellite cell pool, leading to reduced regenerative potential, muscle atrophy, and decline in locomotor activity (Wang et al., 2013). Satellite cells are primarily responsible for muscle regeneration, as in addition to ensuring the preservation of a stem compartment in muscle tissue, they also provide the myogenic cells that will form new myofibrils. Therefore, the loss of the satellite cell pool could reflect the state of muscle alteration that specifically characterizes age-related bone diseases, such as osteoporosis and osteoarthritis (Scimeca et al., 2017).

Sarcopenia is also a condition found in astronauts exposed to spaceflight (di Prampero and Narici, 2003; Bettis et al., 2018). Indeed, prolonged unloading is known to induce rapid tissue degeneration, precluding long-term missions, and reproducing some of the pathophysiological events that occur in the musculoskeletal system during aging (Tarantino et al., 2020). Several scientific evidence have reported that muscle mass and strength may decrease in response to microgravity and ground-based simulation conditions, resulting in muscle atrophy, changes in muscle fiber composition and gene expression, as well as a reduction in regenerative muscle growth (Fitts et al., 2010; Narici and de Boer, 2011; Tanaka et al., 2017). These changes are generally associated with bone mass loss, alterations in the balance between osteoblasts and osteoclasts activities, as well as increased bone resorption and inhibition of new bone formation by osteoblasts (Arfat et al., 2014; Grimm et al., 2016). In this regard, Nabavi and colleagues have subjected primary cultures of murine osteoblasts to 5 days of microgravity, observing important structural alterations, such as shorter microtubules, reduction in the number and size of focal adhesions, and, in general, an extended cell morphology with damaged nuclei (Nabavi et al., 2011). In agreement, Colaianni et al. reported hindlimb unloading as a factor responsible for inducing bone loss and muscle atrophy in mouse models (Colaianni et al., 2017). However, the authors reported that administration of recombinant irisin reversed the effects induced by unload simulation in mice, indicating the potential reversibility of this condition, and suggesting a possible strategy to counteract disease progression (Colaianni et al., 2017). Finally, in our previous work, we evaluated the simulated biological effects of microgravity on primary satellite cell cultures derived from control (CTRL), osteoarthritic (OA), and osteoporotic (OP) patients, finding alterations in both morphological and molecular terms (Tarantino et al., 2020). Specifically, random positioning machine (RPM) exposure resulted in the onset of obvious signs of cell degeneration, such as cytoplasmic vacuolization, and altered the amount of myotubes positive for bone morphogenetic protein-2 (BMP-2) and myostatin. We observed increased BMP-2 expression levels in all experimental groups, whereas myostatin expression was markedly increased in cells from CTRL and OA patients, suggesting the existence of an imbalance between BMP-2 and myostatin pathways (Tarantino et al., 2020).

Not surprisingly, the sarcopenic condition is known to be characterized by a progressive increase in the levels of myostatin, a myokine produced by skeletal muscle on which it acts as a negative regulator (Tarantino et al., 2015b). Indeed, in myostatin-deficient mouse models, an increase in muscle mass and strength has been found, along with an expansion of muscle insertion sites in the humerus, femur, and spine (Elkasrawy and Hamrick, 2010). Qin et al. proposed an anti-osteogenic role for myostatin, having observed that, in response to it, osteocytes increase the expression of sclerostin, Receptor Activator of Nuclear factor Kappa-Β Ligand (RANKL), and Dickkopf-1 (DKK-1), which are all negative regulators of bone remodeling (Qin et al., 2017). In agreement, we recently evaluated myostatin and BMP-2 levels in muscle biopsies from CTRL, OA, and OP patients (Scimeca et al., 2015). Interestingly, the number of myostatin-positive fibers in OP patients was significantly higher than in the CTRL and OA groups, and BMP-2-positive fibers were negative for myostatin and vice versa. In addition, fewer satellite cells were found in the muscles of OP patients than in CTRL and OA patients, confirming a reduction in muscle regenerative potential. Our results suggested that the muscle tissue of OP patients was characterized by a higher content of atrophic fibers, a low amount of satellite cells, and a higher expression of myostatin (Scimeca et al., 2015).

Based on this evidence, in the present work, which is a continuation of the previous one (Tarantino et al., 2020), we reproduced identical experimental conditions and enriched the data previously obtained to evaluate the efficacy of anti-myostatin (anti-MSTN) antibodies in preventing human satellite cell degeneration induced by RPM exposure. Particularly, RPM is a model currently used for simulation research on cell cultures in parallel with experiments conducted on space stations and allows simulating the biological effects of microgravity (van Loon, 2007; Wuest et al., 2015).

## Materials and Methods

### Patients

A total of 30 patients admitted to the Orthopaedic Department of “Tor Vergata” University Hospital were enrolled in this study, excluding all subjects with history of cancer, alcohol abuse, diabetes, myopathies or other neuromuscular diseases or chronic administration of corticosteroid for autoimmune diseases (more than 1 month), viral chronic infections (such as hepatitis B virus—HBV, hepatitis C virus—HCV, and human immunodeficiency virus—HIV), and previously surgical implants. Patients were divided into three groups: 10 patients underwent hip arthroplasty for high-energy hip fracture (CTRL), 10 patients underwent hip arthroplasty for osteoarthritis (OA), and 10 patients underwent hip arthroplasty for fragility fracture (OP). Classification of patients into OA and OP was done according to DEXA, 𝑇-score, and radiographic assessment with the Kellgren–Lawrence scale (Nuti et al., 2019). Patients who underwent hip arthroplasty for high-energy hip fracture were considered as a CTRL group because, in contrast to OA and OP patients, they did not suffer from musculoskeletal system diseases typical of aging, such as osteoarthritis and osteoporosis.

### Bone Mineral Density Evaluation

Dual-energy X-ray absorptiometry (DXA) with a Lunar DXA apparatus (GE Healthcare, Madison, WI, United States) was used to scan the lumbar spine (L1–L4) and femoral (neck and total). According to the manufacturer’s recommendations, BMD was measured in grams per square centimeter, with a coefficient of variation of 0.7% (Celi et al., 2013). BMD was measured on the uninjured limb for patients with fragility fractures; whereas, for all the other patients, measurements were performed on the non-dominant side, with the participants supine on an examination table with their limbs slightly abducted (Piccirilli et al., 2014). DXA evaluation was performed 1 day before surgery for OA patients and 1 month after surgery for CTRL and OP patients. The results were expressed as *T-*scores.

### Radiographic Analysis

Hip radiographs of all patients were performed to check the fracture or to assess hip osteoarthritis, according to a standard validated protocol (Kothari et al., 2004). All radiographs were independently evaluated at different times by two orthopedists using the Kellgren and Lawrence (K–L) radiographic atlas. This system, proposed in 1957, allows classification of osteoarthritis severity using five grades: grade 0 if no radiographic features of osteoarthritis are present; grade 1 if there is doubtful joint space narrowing (JSN) and possible osteophytic lipping; grade 2 if there are definite osteophytes and possible JSN on the anteroposterior weight-bearing radiograph; grade 3 if there are multiple osteophytes, definite JSN, sclerosis, possible bone deformity; grade 4 if there are large osteophytes, marked JSN, severe sclerosis, and definite bone deformity (Kellgren and Lawrence, 1957). Patients with a grade of K–L ≥ 2 were considered osteoarthritic.

### Sampling

Muscle biopsies were taken from the superior portion of the vastus lateralis from CTRL patients undergoing hip arthroplasty for high-energy hip fracture, from OA patients undergoing hip arthroplasty for osteoarthritis, and from OP patients undergoing hip arthroplasty for fragility fracture. The collected samples were subsequently processed for setting up of primary satellite cell cultures, microscopic analysis, and immunocytochemistry. Samples were handled and performed according to approved guidelines. In addition, all experimental procedures were approved by the ethics committee of “Policlinico Tor Vergata” (approval reference number #85/12) and carried out according to the Code of Ethics of the World Medical Association (Declaration of Helsinki). Informed consent was obtained from all patients before surgery.

### Isolation and Culture of Primary Human Satellite Cells

Primary cultures of satellite cells were established in monolayers according to Askanas’s protocol (Askanas and Engel, 1975). Muscle tissues were cleaned and transferred to culture dishes with Conditioning Media (CM; Medium 199 containing Earle’s Salts, stable Glutamine, 25 mM HEPES, 42% Fetal Bovine Serum (FBS), and 1.5% Amphotericin B (Biowest, Nuaillé-France)). After incubation at 37°C overnight and addition 25% Human Plasma (HP) to the CM, culture dishes were again incubated for 7 days at 37°C to verify fibroblast growth. Muscle tissues were removed from clotting media and cut into 1 mm pieces, which were placed in gelatin–plasma-coated dishes (1.5% gelatin). After adding F14 medium [DMEM F14 supplemented with 15% FBS and 0.08% Amphotericin B (Biowest, Nuaillé-France), Penicillin–Streptomycin (Sigma Chemical Co., St. Louis, MO, United States), stable Glutamine (Biowest, Nuaillé-France), 0.01 mg/ml human Insulin, 0.05 μg/ml Fibroblast Growth Factor, and 0.01 μg/ml epidermal growth factor], culture dishes were incubated at 37°C for 5–7 days. After substantial growth, tissues were removed, and satellite cells were placed on new culture dishes coated with 0.2% gelatin. As soon as the cells began to fuse into myotubes, they were washed with Hank’s solution without calcium and magnesium and then switched to medium containing F14, 5% FBS, PSF, and human insulin but no growth factor. Cell cultures were fed twice a week until experimental use. After fixation of satellite cells with 4% paraformaldehyde dissolved in 0.9% saline solution for 4 h, two blinded pathologists performed morphological analysis using a Nikon upright microscope ECLIPSE Ci-S/(Nikon Corporation, Tokyo, Japan) connected to a Nikon digital camera. Images were acquired at 4× and 20× magnification using NIS-Elements software (5.30.01; Laboratory Imaging, Prague, Czech Republic).

### Simulation Research by RPM

The RPM system (Airbus Defence and Space Netherlands B.V.) was used to simulate the biological effects of microgravity on satellite cells (Borst and Van Loon, 2009). All experiments were carefully planned according to procedures previously described (Wuest et al., 2015; Tarantino et al., 2020). The rotating RPM frame was placed inside an ordinary cell culture CO_2_ incubator. The software responsible for controlling the motion of RPM employed a tailored algorithm, which rotated with a random speed in such a way that the mean gravity vector reliably converged to zero over time. The samples were positioned compactly in the center of rotation, to minimize centrifugal acceleration and to avoid artifacts. For *in vitro* cultivation, we used 24-well plates sealed with dialysis membrane (Visking Medicell International Ltd., Liverpool Road—London code DTV12000.06.000 MWCO 12/14 Kdalton). Each well was sealed by deposition of a dialysis membrane on the convex liquid meniscus of the medium inside the well to prevent the formation of air bubbles. The nitrocellulose discs were fixed to the support by means of a rubber ring to minimize the effects of flow shear on the attached cells. Primary satellite cell cultures were exposed to RPM for 72 h; while plates exposed to normogravity regime were kept in incubator for the same period, so that all cell samples shared the same experimental conditions.

### Immunostaining of Primary Human Satellite Cells

The expression of the Pax7 by immunofluorescence was evaluated for the characterization of satellite cells from CTRL, OA, and OP patients. After fixation in 4% paraformaldehyde dissolved in 0.9% saline solution for 30 min, cell cultures were pretreated with ethylenediaminetetraacetic acid (EDTA) citrate, pH 7.8 for 20 min at 95°C, and incubated with rabbit monoclonal anti-Pax7 antibodies for 60 min (clone NC, Novus Biologicals). Reaction was revealed by using Texas red anti-rabbit secondary antibodies (Novus Biologicals, Littleton, CO, United Sates). Washing was performed with PBS/Tween20 pH 7.6 (UCS Diagnostic, Rome, Italy). Finally, cells were counteracted with 4′,6-diamidino-2-phenylindole (DAPI) counterstain (Kreatech Biotechnology B.V., Amsterdam, Netherlands).

### Immunocytochemistry

Immunocytochemical characterization was performed on culture dishes after fixation in 4% paraformaldehyde dissolved in 0.9% saline solution for 30 min to assess myostatin expression in all primary cultures of satellite cells (Tarantino et al., 2020). Cell samples were pretreated with EDTA citrate, pH 7.8 for 30 min at 95°C, and then incubated for 1 h with rabbit monoclonal anti-myostatin (clone ab134682, AbCam, Cambridge, United Kingdom). Washings were performed with PBS/Tween20 pH 7.6 (UCS Diagnostic, Rome, Italy); horseradish peroxidase (HRP)-3,3′ diaminobenzidine (DAB) Detection Kit (UCS Diagnostic, Rome, Italy) was used to reveal immunocytochemical reactions. Specifically, 50 μl DAB/450 μl of substrate was incubated for 3 min. To assess the background of immunostaining, we included negative controls for each reaction by incubating the sections with secondary antibodies (HRP) alone or a detection system (DAB) alone. Immunocytochemical positivity was assessed on digital images acquired with NIS-Elements software (5.30.01; Laboratory Imaging, Prague, Czech Republic) using a semi-quantitative approach, scoring from 1 to 3 based on the number of positive myotubes out of the total analyzed for myostatin. Results were shown as percentage of positive myotubes. For each patient, the experiment was conducted in triplicate (*n* = 30/group).

### Primary Cultures of Human Satellite Cells Conditioned With Anti-MSTN Antibodies

The role of myostatin in the activity of primary cultures of human satellite cells exposed to RPM was investigated by their treatment with anti-MSTN antibodies (clone ab134682, AbCam, Cambridge, United Kingdom). Specifically, cells from the first or second passage were seeded in a 24-well plate at a density of 10 × 10^3^ cells/well. Primary cultures derived from each patient group were incubated with 1 μg/ml anti-MSTN antibodies and exposed to RPM for 72 h. The control experiment was performed by injecting a 0.9% saline solution (Vehicle) into the primary cultures of the same patients also subjected to 72 h of RPM exposure. At the end of the experiment, cell cultures were fixed in 4% paraformaldehyde dissolved in 0.9% saline solution for 30 min and subjected to Toluidine blue staining to study cell proliferation and morphology.

### Statistical Analysis

Statistical analysis was performed using GraphPad Prism 8 Software (Prism 8.0.1, La Jolla, CA, United States). Clinical data were analyzed by the Mann–Whitney test. For immunocytochemistry, data were expressed as mean ± SEM, and *n* represents the percentage of myostatin-positive myotubes number. Data were compared with one-way ANOVA and Tukey’s multiple comparison test and were considered significantly different if *p* < 0.05.

## Results

### Clinical Evaluation

Characterization of all patients enrolled in the study was performed by clinical and instrumental assessment. As shown in Table 1, we analyzed the following parameters for each group of patients: age (years), Bone Mass Index (BMI), *T-*score L1–L4, *T-*score (femoral neck), and *T-*score (total femur). Specifically, the CTRL group included 10 patients characterized by a *T-*score ≥ −1.0 SD and a K–L score from 0 to 1. The OA group included 10 patients with radiographic evidence of hip OA, a K–L score of 3 or 4, and a *T-*score ≥ −2.5 SD; whereas the OP group included 10 patients with fragility hip fracture, *T-*score ≤ −2.5 SD, and a K–L score from 0 to 1. We found no discrepancy for age, sex, and comorbidities between the OA (mean age in years: 72.5 ± 3.2) and OP (mean age in years: 78.4 ± 2.5) groups, compared with CTRL patients (mean age in years: 45.3 ± 2.6) who were significantly younger (^***^p < 0.001). In contrast, we found significant differences between the three groups for the other parameters analyzed (BMI, ^*^p < 0.05; *T-*score, ^***^p < 0.001). Not surprisingly, the CTRL group presented higher *T-*score values than the OA and OP patients, highlighting a dependence of musculoskeletal disorders on patient age.

**Table 1:** Main characteristics of CTRL, OA, and OP patients.

	Age (years)	BMI (Kg/m^2^)	*T-*score (L1–L4)	*T-*score (femoral neck)	*T-*score (total femur)
CTRL	45.3 ± 2.6	23.1 ± 2.9	0.9 ± 0.5	1.2 ± 0.1	1.0 ± 0.3
OA	72.5 ± 3.2	27.3 ± 1.0	−0.7 ± 0.6	−0.6 ± 0.1	−0.4 ± 0.2
OP	78.4 ± 2.5	21.4 ± 1.5	−2.5 ± 0.4	−2.7 ± 0.2	−2.4 ± 0.1

*CTRL, patients underwent hip arthroplasty for high-energy hip fracture; OA, patients underwent hip arthroplasty for osteoarthritis; OP, patients underwent hip arthroplasty for fragility fracture; BMI, Bone Mass Index*.

### Morphological Analysis

Morphological analysis by light microscopy was performed to assess cellular composition and to verify that all primary cultures were free of contamination by pathogens, such as bacteria, algae, and fungi, and/or cells of different histo-types, such as fibroblasts. In this regard, the Pax7 expression by immunofluorescence was assessed to characterize the satellite cell population (Supplementary Figure S1).

**Figure 1:**
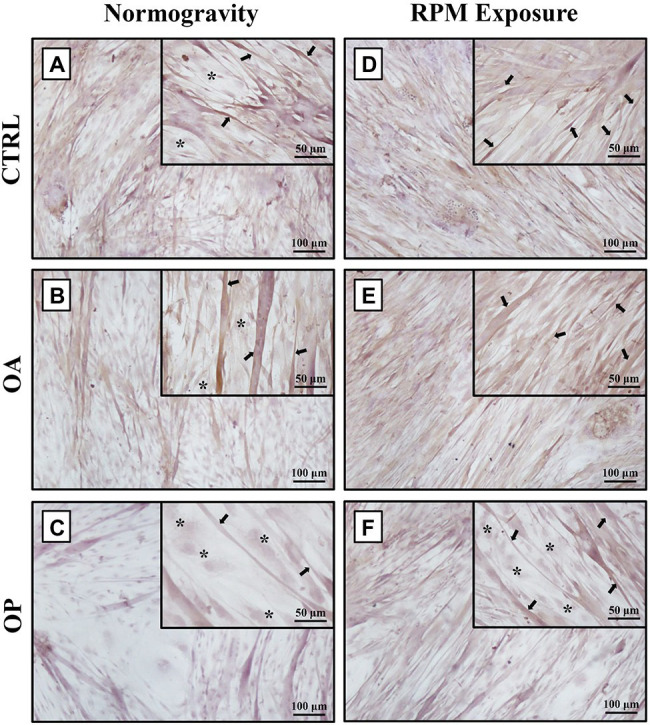
Morphological analysis by light microscopy of primary cultures of human satellite cells subjected to normogravity regimen and RPM exposure. In normogravity conditions, primary cultures from CTRL **(A)** and OA **(B)** patients were characterized by a heterogeneous population of numerous myotubes (arrows) and rare single satellite cells (asterisks), whereas primary cultures from OP patients **(C)** consisted of rare myotubes (arrows) and numerous single satellite cells (asterisks). RPM exposure caused an increase in the number of myotubes (arrows) in primary cultures from CTRL **(D)** and OA **(E)** patients. Samples derived from OP patients **(F)** showed not only single satellite cells (asterisks), but also several myotubes (arrows). Satellite cells (asterisks) were characterized by immunofluorescence for Pax7 (see Supplementary Figure S1). 4× images, scale bar represents 100 μm; 20× images, scale bar represents 50 μm.

In agreement with previous results (Tarantino et al., 2020), under normogravity conditions, samples derived from CTRL (Figure 1A) and OA (Figure 1B) patients showed similar morphological characteristics, with a heterogeneous population of numerous myotubes and rare single satellite cells. In contrast, in primary cultures from OP patients (Figure 1C), we observed numerous single satellite cells and rare myotubes. Noteworthy, cell morphology was affected by RPM exposure, as variations in the satellite cell/myotube ratio were found in all three experimental groups (Figures 1D–F). However, although an increase in the number of myotubes was observed mainly in OP patients, microscopic evaluation also showed that RPM exposure induced cell degeneration, as demonstrated by cytoplasmic vacuolization of myotubes and the presence of numerous cellular debris and areas of necrosis.

### Immunocytochemistry

Myostatin analysis was performed by immunocytochemistry, and the levels of its expression were quantified by counting the number of positive myotubes over 25 High-Power Field.

**Figure 2:**
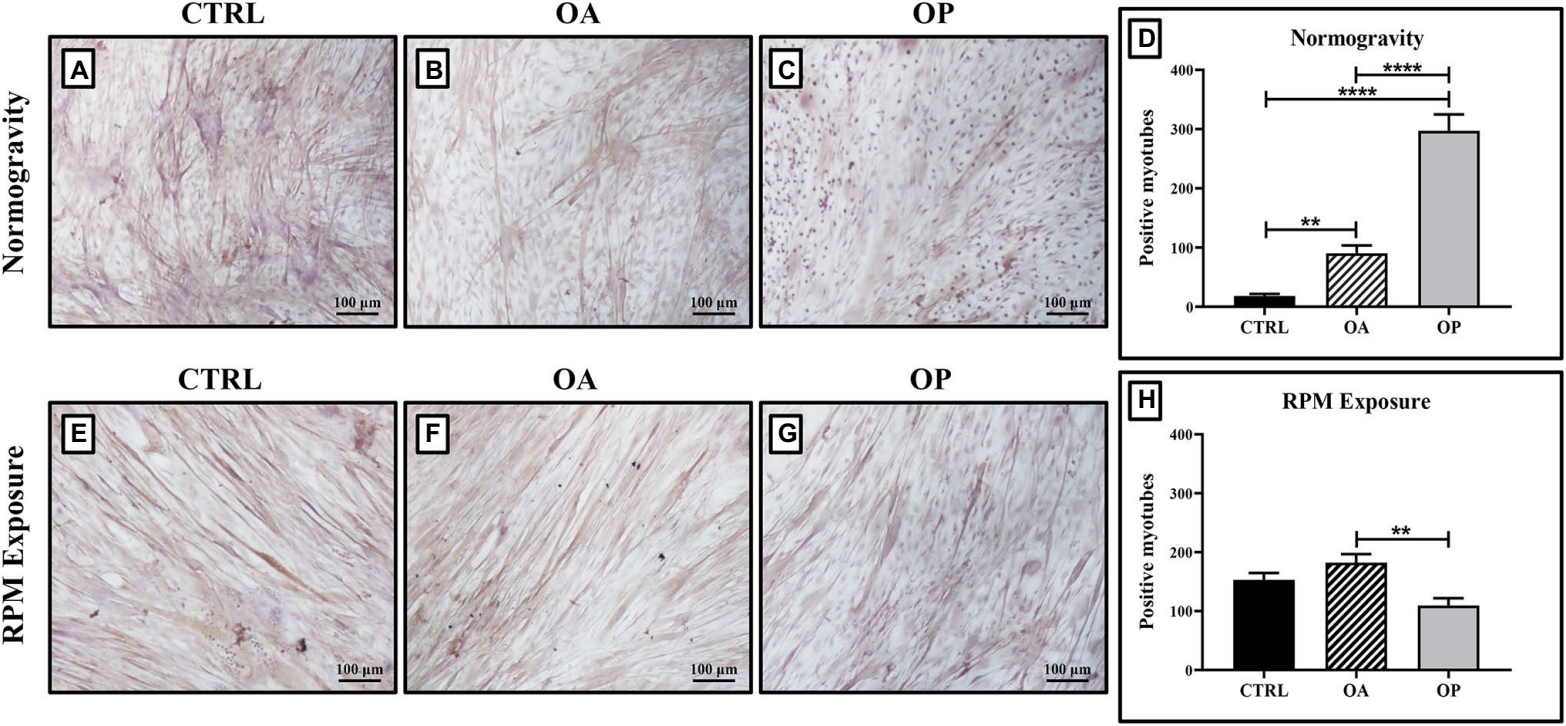
Evaluation of myostatin expression by immunocytochemistry. In cell cultures exposed to normogravity regimen, a significant increase in myostatin expression was observed in OA **(B)** and OP **(C)** patients respect to the CTRL group **(A)**. Note that a significant statistical difference was reported between the following groups **(D)**: CTRL vs. OA, ^**^p < 0.01; CTRL vs. OP, ^****^p < 0.0001; OA vs. OP, ^****^p < 0.0001. After RPM exposure, primary cultures derived from OP patients **(G)** showed a significant reduction in myostatin expression, whereas higher levels of myostatin were found in CTRL **(E)** and OA **(F)** patients. Note that a significant statistical difference was found only between the OA and OP groups (OA vs. OP, ^**^p < 0.01; **H**). 4x images, scale bar represents 100 μm.

In normogravity conditions, primary cultures derived from OP patients showed the highest levels of myostatin expression compared with the CTRL and OA groups. A significant increase in myostatin expression was also observed in primary cultures derived from OA patients compared with CTRL patients (Figures 2A–C). Indeed, myostatin levels were (18.1 ± 2.4) in the CTRL group, (90.3 ± 8.4) in the OA group, and (295.9 ± 14.5) in the OP group (CTRL vs. OA, ^**^p < 0.01; CTRL vs. OP, ^****^p < 0.0001; OA vs. OP, ^****^p < 0.0001; Figure 2D). These data do not differ from those previously shown (Tarantino et al., 2020), confirming that myostatin expression is age-dependent. RPM exposure did not induce changes in myostatin expression between CTRL and OA groups, in contrast to cells derived from OP patients in which myostatin levels were significantly reduced (Figures 2E–G). Thus, we observed group-dependent myostatin expression, with values of (153.1 ± 11.3) for CTRL patients, (182.2 ± 15.4) for OA patients, and (109.7 ± 8.1) for OP patients (OA vs. OP, ^**^p < 0.01; Figure 2H).

### Anti-MSTN Antibodies Treatment on Primary Cultures of Human Satellite Cells

We previously showed by Transmission Electron Microscopy (TEM) analysis the presence of evident signs of cell degeneration in myotubes observed in primary cultures subjected to RPM exposure (Tarantino et al., 2020). To investigate whether myostatin was the main effector of such degeneration, we treated satellite cells exposed to RPM with anti-MSTN antibodies for 72 h. Toluidine blue staining allowed us to evaluate important morphological differences between the three groups of patients under the two experimental conditions.

**Figure 3:**
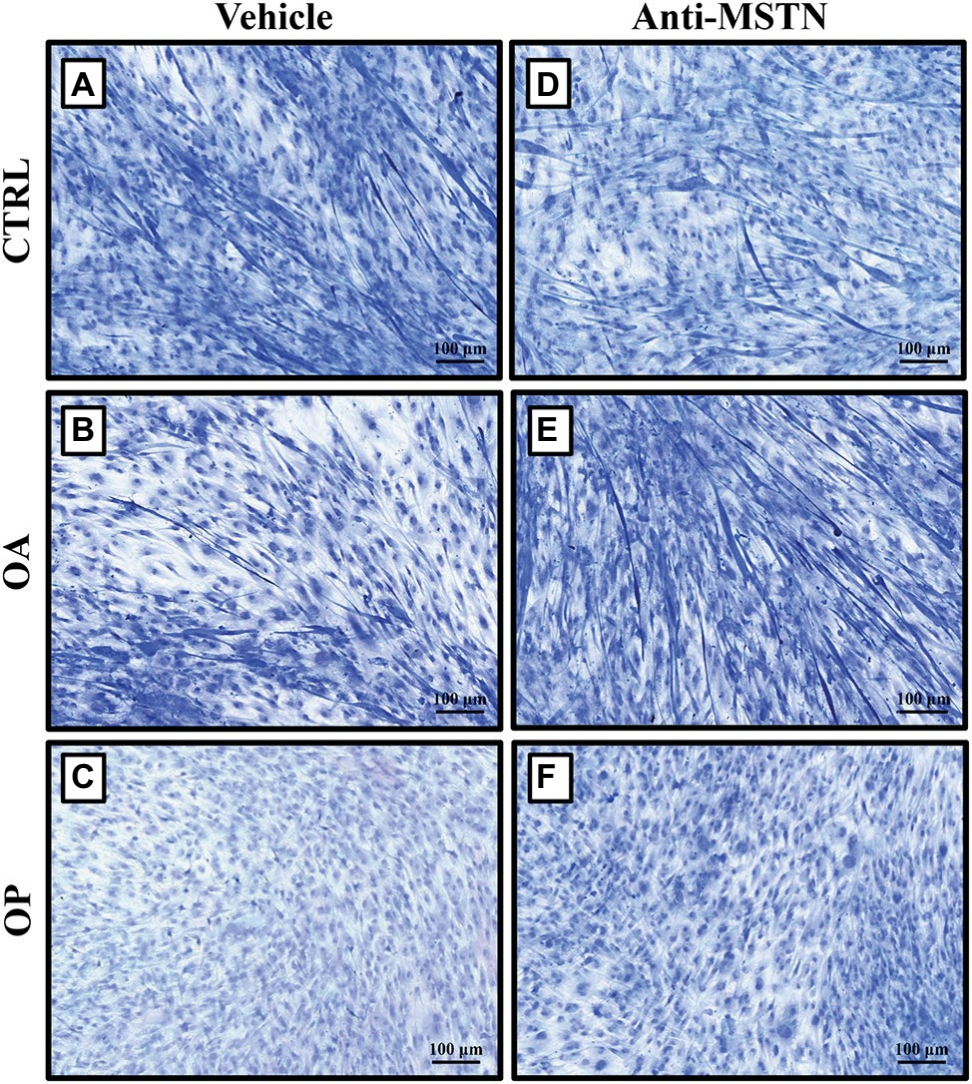
Effects of anti-MSTN antibodies on primary cultures of human satellite cells subjected to RPM exposure. **(A)** In CTRL patients, myotubes subjected to RPM exposure showed slowed growth; whereas treatment with anti-MSTN antibodies **(D)** allowed to counteract this negative effect, promoting in some places the formation of a cellular multilayer. **(B)** In OA patients, myotubes subjected to RPM exposure exhibited slowed growth; treatment with anti-MSTN antibodies **(E)** induced growth and formation of new myotubes. **(C)** In OP patients, RPM exposure dramatically reduced the ability of cells to form myotubes; while treatment with anti-MSTN antibodies **(F)** promoted cell aggregation and the tendency to form new myotubes even after RPM exposure. 10x images, scale bar represents 100 μm.

Regarding to CTRL patients, we found a slowing of growth in myotubes subjected to RPM exposure (Figure 3A). On the contrary, treatment with anti-MSTN antibodies counteracted this negative effect, favoring in some places the formation of a cellular multilayer (Figure 3D). Also in OA patients, myotubes subjected to RPM exposure showed slowed growth (Figure 3B); whereas treatment with anti-MSTN antibodies promoted growth and formation of new myotubes (Figure 3E). Finally, RPM exposure drastically impaired the ability to form myotubes in OP patient cultures (Figure 3C). Surprisingly, even in this experimental group, treatment with anti-MSTN antibodies counteracted the deleterious effects of RPM exposure by promoting cell aggregation and the tendency to form new myotubes (Figure 3F).

## Discussion

In our previous work, primary cultures of human satellite cells provided to be a useful scientific model to identify biomolecular processes involved in muscle mass loss related to alteration of normal mechanical loading (Tarantino et al., 2020). Indeed, prolonged exposure to no load is known to affect regenerative growth and tissue repair by impairing adult stem cell proliferation and differentiation processes (Blaber et al., 2014). The negative impact of microgravity and ground-based simulation conditions on physiological systems has been confirmed by numerous studies, which report skeletal muscle cell atrophy, inhibition of osteoblast differentiation, reduction in osteoblast number, altered immune cell activation, and abnormal chondrocyte formation as the main harmful effects on cellular function (Nabavi et al., 2011; Chatziravdeli et al., 2019; Wehland et al., 2020; Ludtka et al., 2021). Based on this evidence and taking our previous work as a starting point (Tarantino et al., 2020), we have here evaluated the efficacy of anti-MSTN antibodies in preventing muscle degeneration induced by RPM exposure in primary cultures of human satellite cells, reproducing identical experimental conditions and enriching previously obtained data.

Sarcopenia, a major age-related musculoskeletal disorder, is characterized by quantitative and qualitative changes in the structure and function of the musculoskeletal system. This process is typically slow but, at the same time, inevitable, as it occurs even in healthy, well-nourished, and physically active individuals (Korhonen et al., 2006). Although the biological processes underlying aging have not yet been fully elucidated, several alterations have been observed in aging muscle tissue. For example, in old age there is a significant reorganization of the fibers that make up motor units, with an increase in their size but a reduction in their number. In addition, during the aging process there is a progressive loss of motor neurons, leading to a decline in the neuromuscular system (Larsson et al., 2019). These alterations are associated with others that promote the sarcopenia progression, such as altered microcirculation, mitochondrial damage, and loss of regenerative potential (Ryan et al., 2006; Degens, 2010; Xu et al., 2021). The muscle decline strongly affects bone tropism, mobility, and skeletal system characteristics (Scimeca et al., 2015). Noteworthy, recent studies have reported a strong association between sarcopenia and the occurrence of degenerative bone diseases, such as osteoporosis and osteoarthritis, suggesting a key role of bone–muscle crosstalk in understanding the pathophysiology of sarcopenia (Tarantino et al., 2015a; Cariati et al., 2021). Therefore, in this study we included OA and OP patients undergoing hip arthroplasty for osteoarthritis and fragility fracture, respectively. In addition, for the CTRL group, we enrolled young patients who had undergone hip arthroplasty for a high-energy fracture but did not suffer from age-related musculoskeletal disorders. During surgery, biopsies were taken from the vastus lateralis muscle of each patient, to set up primary satellite cell cultures used at first or second passage.

First, satellite cells were characterized by immunofluorescence to assess the expression of Pax7, a key transcriptional regulator expressed by quiescent satellite cells. Then, we performed a morphological analysis by light microscopy to verify the presence of any peculiar features in the three experimental groups. In normogravity conditions, CTRL and OA groups presented a similar appearance, as both cell culture types were characterized by a heterogeneous population of numerous myotubes and rare single satellite cells. In contrast, samples derived from OP patients consisted mostly of single satellite cells and rare myotubes. These results are in line with our previous study conducted on muscle biopsies from OA and OP patients, in which immunohistochemistry showed a higher expression of Pax7 and a better ability to form new fibers in the muscle of OA patients compared to the OP group (Tarantino et al., 2016). In agreement with previous data (Tarantino et al., 2020), RPM exposure influenced cell morphology, as variations in the satellite cell/myotube ratio were found in the three experimental groups. Particularly, a significant increase in the number of myotubes was observed in the OP group.

As demonstrated by Scimeca et al. (2017), the imbalance between the bone morphogenetic proteins (BMPs) pathway, which plays a role in controlling the mass and regeneration of adult skeletal muscle, and the myostatin pathway, which acts as a potent negative regulator of muscle growth, could be responsible for the muscle degeneration that characterizes the sarcopenic condition (Scimeca et al., 2017). Specifically, *in vitro* studies have shown that myostatin blocks myoblast proliferation and satellite cell self-renewal by downregulation of MyoD, resulting in inhibition of myogenesis (Thomas et al., 2000). Moreover, it competes for both BMP receptor binding and Smad4 activation, suggesting that muscle quality is strongly influenced by the balance between myostatin and BMPs pathways (Snijders et al., 2015). Based on these evidence, we subjected primary satellite cell cultures to immunocytochemical analysis to assess differences in myostatin expression between the three experimental groups. Not surprisingly, its expression was age-dependent under normogravity conditions, as the lowest myostatin levels were found in CTRL patients, who had a mean age of 45.3 ± 2.6, whereas the OP group, with a mean age of 78.4 ± 2.5, had the highest myostatin levels. RPM exposure induced group-dependent changes in myostatin expression. Specifically, we observed similar levels in myostatin expression in CTRL and OA groups, although with higher values than those measured in normogravity conditions. Surprisingly, a significant reduction in myostatin levels was detected in cell cultures derived from OP patients, with far lower values when compared to those measured under normogravity regime. However, this reduction could be a transient effect due to the mechanical insult, as observed in our previous work, in which TEM analysis showed that RPM exposure induces phenomena of degeneration and cell death in all experimental groups, including the presence of lipofuscin granules, electron dense cytoplasm, and dystrophic calcifications (Tarantino et al., 2020).

Considering the results obtained from morphological evaluation and immunocytochemistry, we hypothesized that myostatin might be the main effector of such degeneration. Therefore, to test whether inactivation of the myostatin pathway was preparatory to blocking/delaying the negative effects of RPM exposure on satellite cells, we subjected primary cultures to treatment with anti-MSTN antibodies for 72 h and then to Toluidine blue staining for morphological analysis. Surprisingly, a significant increase in cell survival was observed in cultures exposed to RPM and treated with anti-MSTN antibodies, in all experimental groups. In detail, myostatin inhibition induced the formation of a pluristrate of myotubes in both CTRL and OA patients. Furthermore, in cell cultures derived from the OP group, where the ability to form myotubes was drastically inhibited, treatment with anti-MSTN antibodies promoted cell aggregation and the tendency to form myotubes even after RPM exposure.

## Limits of the Study

The main limit of this study is the age difference of the CTRL group compared to OA and OP patients, which undoubtedly affects their clinical characteristics, such as BMI and *T-*score. Unfortunately, it is not easy to enroll age-matched subjects of OA and OP patients, who do not have musculoskeletal disorders, but have undergone hip arthroplasty for high-energy fracture. However, in our research experience we have frequently observed similar responses between the samples of CTRL and OA patients. Another limit of our study is represented by the RPM, the use of which could underestimate or overestimate the effects of no load on biological samples. In fact, the influence of the effects of flow shear on the experimental result should not be neglected, ensuring that the culture chamber is free of air bubbles responsible for detachment from the substrate and consequent cell death. Therefore, results obtained from experiments using RPM systems to simulate of the biological effects of microgravity should be interpreted with caution and, if possible, compared directly with experiments under real microgravity conditions.

## Conclusion

**Figure 4:**
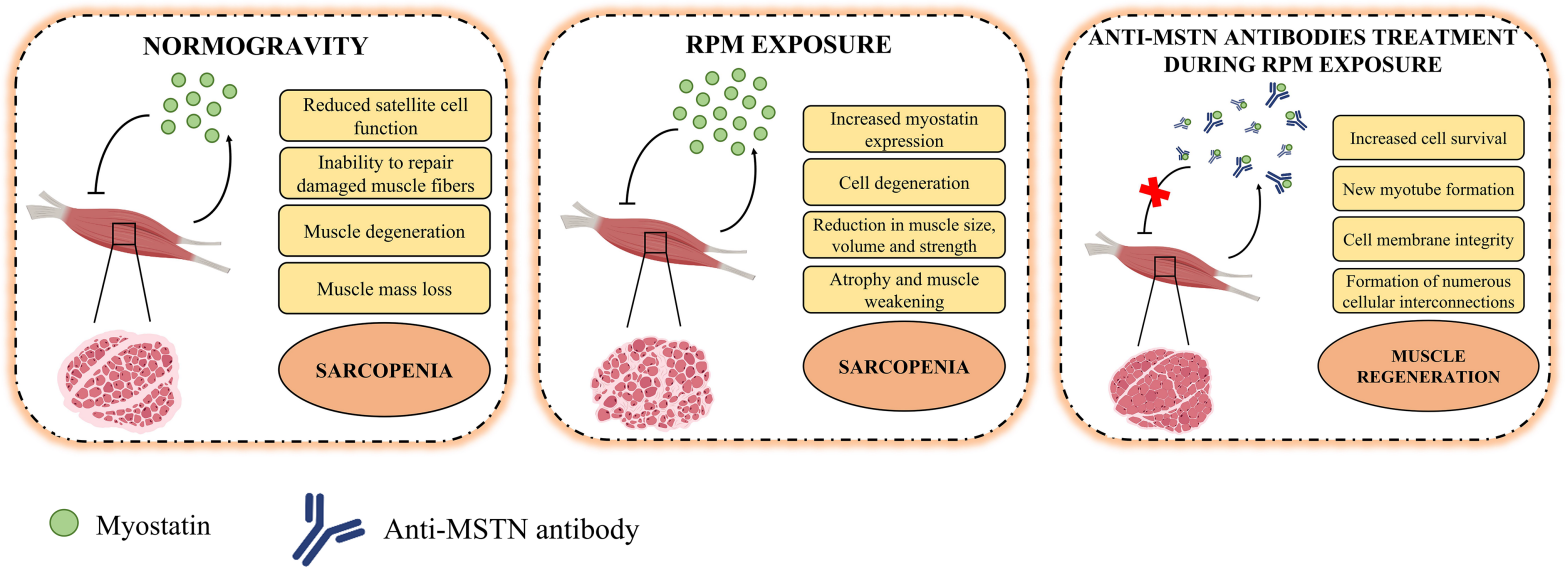
Role of myostatin in the pathophysiology of muscle tissue. In normogravity, myostatin is produced by skeletal muscle on which it acts as a negative regulator, reducing satellite cell function and their ability to repair damaged muscle fibers, inducing degeneration and muscle mass loss, and so promoting the progression of sarcopenia. After RPM exposure, the increases myostatin expression caused cell degeneration and reduction in muscle size, volume, and strength. These conditions amplified atrophy and muscle weakening and accelerated the sarcopenia progression. The anti-MSTN antibodies treatment blocks the action of myostatin and promote an increase in cell survival, the formation of new myotubes and muscle regeneration.

The results presented here support the hypothesis of myostatin-mediated muscle damage induced by RPM exposure. In fact, the action of this negative regulator on skeletal muscle was counteracted by the treatment of satellite cells with anti-MSTN antibodies (Figure 4). However, the reversibility of damage induced by RPM exposure was observed for a period of 72 h, suggesting the need for further studies with longer exposure times to assess the limit of damage reversibility. Furthermore, the role of potential other effectors responsible for muscle degeneration by RPM exposure will need to be investigated to understand their involvement in the sarcopenic condition development. Finally, although RPM represents a valid model for simulating the biological effects of microgravity for us, it is not the only strategy capable of reproducing the effects induced by no load. Therefore, preclinical studies on the use of anti-MSTN antibodies in model animals subjected to limb unloading will be needed to confirm and validate the efficacy of this treatment in counteracting the loss of muscle mass typical of sarcopenia.

## Data Availability Statement

The raw data supporting the conclusions of this article will be made available by the authors, without undue reservation.

## Ethics Statement

All sampling and experiments described in the present study were performed in agreement with independent ethical committee of “Policlinico Tor Vergata” (approval reference number 85/12). All experimental procedures were carried out according to the Code of Ethics of the World Medical Association (Declaration of Helsinki). The patients/participants provided their written informed consent to participate in this study.

## Author Contributions

IC, MS, and UT developed the hypotheses and designed the experimental plan. IC, RB, MS, and MM performed and analyzed the experiments and contributed to the interpretation of the data. IC and RB wrote and edited the manuscript. RT, VN, GT, and RI contributed to the data curation. VT, EG, and UT assisted in drafts and final version of the manuscript. All authors read and approved the final manuscript.

## Funding

This research was funded by BRIC-INAIL (2019#23).

## Conflict of Interest

The authors declare that the research was conducted in the absence of any commercial or financial relationships that could be construed as a potential conflict of interest.

## Publisher’s Note

All claims expressed in this article are solely those of the authors and do not necessarily represent those of their affiliated organizations, or those of the publisher, the editors and the reviewers. Any product that may be evaluated in this article, or claim that may be made by its manufacturer, is not guaranteed or endorsed by the publisher.

## References

[ref1] Alway S. E. Siu P. M. (2008). Nuclear apoptosis contributes to sarcopenia. Exerc. Sport Sci. Rev. 36, 51–57. doi: 10.1097/JES.0b013e318168e9dc, PMID: 18362685 PMC2778230

[ref2] Arfat Y. Xiao W.-Z. Iftikhar S. Zhao F. Li D.-J. Sun Y.-L.. (2014). Physiological effects of microgravity on bone cells. Calcif. Tissue Int. 94, 569–579. doi: 10.1007/s00223-014-9851-x, PMID: 24687524

[ref3] Askanas V. Engel W. K. (1975). A new program for investigating adult human skeletal muscle grown aneurally in tissue culture. Neurology 25, 58–67. doi: 10.1212/wnl.25.1.58, PMID: 46113

[ref4] Bettis T. Kim B.-J. Hamrick M. W. (2018). Impact of muscle atrophy on bone metabolism and bone strength: implications for muscle-bone crosstalk with aging and disuse. Osteoporos. Int. 29, 1713–1720. doi: 10.1007/s00198-018-4570-1, PMID: 29777277 PMC7861141

[ref5] Blaber E. Sato K. Almeida E. A. C. (2014). Stem cell health and tissue regeneration in microgravity. Stem Cells Dev. 23(Suppl. 1), 73–78. doi: 10.1089/scd.2014.0408, PMID: 25457968 PMC4235978

[ref6] Borst A. G. Van Loon J. J. W. A. (2009). Technology and developments for the random positioning machine, RPM. Microgravity Sci. Technol. 21, 287–292. doi: 10.1007/s12217-008-9043-2

[ref7] Cariati I. Bonanni R. Onorato F. Mastrogregori A. Rossi D. Iundusi R.. (2021). Role of physical activity in bone-muscle crosstalk: biological aspects and clinical implications. J. Funct. Morphol. Kinesiol. 6:55. doi: 10.3390/jfmk6020055, PMID: 34205747 PMC8293201

[ref8] Celi M. Rao C. Scialdoni A. Tempesta V. Gasbarra E. Pistillo P.. (2013). Bone mineral density evaluation in osteoporosis: why yes and why not? Aging Clin. Exp. Res. 25(Suppl. 1), 47–49. doi: 10.1007/s40520-013-0074-1, PMID: 24046042

[ref9] Chatziravdeli V. Katsaras G. N. Lambrou G. I. (2019). Gene expression in osteoblasts and osteoclasts under microgravity conditions: a systematic review. Curr. Genomics 20, 184–198. doi: 10.2174/1389202920666190422142053, PMID: 31929726 PMC6935951

[ref10] Colaianni G. Mongelli T. Cuscito C. Pignataro P. Lippo L. Spiro G.. (2017). Irisin prevents and restores bone loss and muscle atrophy in hind-limb suspended mice. Sci. Rep. 7:2811. doi: 10.1038/s41598-017-02557-8, PMID: 28588307 PMC5460172

[ref11] Degens H. (2010). The role of systemic inflammation in age-related muscle weakness and wasting. Scand. J. Med. Sci. Sports 20, 28–38. doi: 10.1111/j.1600-0838.2009.01018.x, PMID: 19804579

[ref12] Deschenes M. R. (2004). Effects of aging on muscle fibre type and size. Sports Med. 34, 809–824. doi: 10.2165/00007256-200434120-00002, PMID: 15462613

[ref13] di Prampero P. E. Narici M. V. (2003). Muscles in microgravity: from fibres to human motion. J. Biomech. 36, 403–412. doi: 10.1016/s0021-9290(02)00418-9, PMID: 12594988

[ref14] Elkasrawy M. N. Hamrick M. W. (2010). Myostatin (GDF-8) as a key factor linking muscle mass and bone structure. J. Musculoskelet. Neuronal Interact. 10, 56–63. PMID: 20190380 PMC3753581

[ref15] Fitts R. H. Trappe S. W. Costill D. L. Gallagher P. M. Creer A. C. Colloton P. A.. (2010). Prolonged space flight-induced alterations in the structure and function of human skeletal muscle fibres. J. Physiol. 588, 3567–3592. doi: 10.1113/jphysiol.2010.188508, PMID: 20660569 PMC2988519

[ref16] Grimm D. Grosse J. Wehland M. Mann V. Reseland J. E. Sundaresan A.. (2016). The impact of microgravity on bone in humans. Bone 87, 44–56. doi: 10.1016/j.bone.2015.12.057, PMID: 27032715

[ref17] Kellgren J. H. Lawrence J. S. (1957). Radiological assessment of osteo-arthrosis. Ann. Rheum. Dis. 16, 494–502. doi: 10.1136/ard.16.4.494, PMID: 13498604 PMC1006995

[ref18] Korhonen M. T. Cristea A. Alén M. Häkkinen K. Sipilä S. Mero A.. (2006). Aging, muscle fiber type, and contractile function in sprint-trained athletes. J. Appl. Physiol. 101, 906–917. doi: 10.1152/japplphysiol.00299.2006, PMID: 16690791

[ref19] Kothari M. Guermazi A. von Ingersleben G. Miaux Y. Sieffert M. Block J. E.. (2004). Fixed-flexion radiography of the knee provides reproducible joint space width measurements in osteoarthritis. Eur. Radiol. 14, 1568–1573. doi: 10.1007/s00330-004-2312-6, PMID: 15150666

[ref20] Larsson L. Degens H. Li M. Salviati L. Lee Y. I. Thompson W.. (2019). Sarcopenia: aging-related loss of muscle mass and function. Physiol. Rev. 99, 427–511. doi: 10.1152/physrev.00061.2017, PMID: 30427277 PMC6442923

[ref21] Ludtka C. Silberman J. Moore E. Allen J. B. (2021). Macrophages in microgravity: the impact of space on immune cells. npj Microgravity 7:13. doi: 10.1038/s41526-021-00141-z, PMID: 33790288 PMC8012370

[ref22] Marzetti E. Calvani R. Cesari M. Buford T. W. Lorenzi M. Behnke B. J.. (2013). Mitochondrial dysfunction and sarcopenia of aging: from signaling pathways to clinical trials. Int. J. Biochem. Cell Biol. 45, 2288–2301. doi: 10.1016/j.biocel.2013.06.024, PMID: 23845738 PMC3759621

[ref23] Nabavi N. Khandani A. Camirand A. Harrison R. E. (2011). Effects of microgravity on osteoclast bone resorption and osteoblast cytoskeletal organization and adhesion. Bone 49, 965–974. doi: 10.1016/j.bone.2011.07.036, PMID: 21839189

[ref24] Narici M. V. de Boer M. D. (2011). Disuse of the musculo-skeletal system in space and on earth. Eur. J. Appl. Physiol. 111, 403–420. doi: 10.1007/s00421-010-1556-x, PMID: 20617334

[ref25] Nuti R. Brandi M. L. Checchia G. Di Munno O. Dominguez L. Falaschi P.. (2019). Guidelines for the management of osteoporosis and fragility fractures. Intern. Emerg. Med. 14, 85–102. doi: 10.1007/s11739-018-1874-2, PMID: 29948835 PMC6329834

[ref26] Piccirilli E. Gasbarra E. Baldi J. Pistillo P. Tarantino U. (2014). Can muscular impairment be the key for femoral fracture? J. Gerontol. Geriatr. Res. 03, 3–6. doi: 10.4172/2167-7182.1000183

[ref27] Qin Y. Peng Y. Zhao W. Pan J. Ksiezak-Reding H. Cardozo C.. (2017). Myostatin inhibits osteoblastic differentiation by suppressing osteocyte-derived exosomal microRNA-218: a novel mechanism in muscle-bone communication. J. Biol. Chem. 292, 11021–11033. doi: 10.1074/jbc.M116.770941, PMID: 28465350 PMC5491785

[ref28] Ryan N. A. Zwetsloot K. A. Westerkamp L. M. Hickner R. C. Pofahl W. E. Gavin T. P. (2006). Lower skeletal muscle capillarization and VEGF expression in aged vs. young men. J. Appl. Physiol. 100, 178–185. doi: 10.1152/japplphysiol.00827.2005, PMID: 16166239

[ref29] Scimeca M. Bonanno E. Piccirilli E. Baldi J. Mauriello A. Orlandi A.. (2015). Satellite cells CD44 positive drive muscle regeneration in osteoarthritis patients. Stem Cells Int. 2015:469459. doi: 10.1155/2015/469459, PMID: 26101529 PMC4460235

[ref30] Scimeca M. Piccirilli E. Mastrangeli F. Rao C. Feola M. Orlandi A.. (2017). Bone morphogenetic proteins and myostatin pathways: key mediator of human sarcopenia. J. Transl. Med. 15:34. doi: 10.1186/s12967-017-1143-6, PMID: 28202082 PMC5310081

[ref31] Snijders T. Nederveen J. P. McKay B. R. Joanisse S. Verdijk L. B. van Loon L. J. C.. (2015). Satellite cells in human skeletal muscle plasticity. Front. Physiol. 6:283. doi: 10.3389/fphys.2015.00283, PMID: 26557092 PMC4617172

[ref32] Tanaka K. Nishimura N. Kawai Y. (2017). Adaptation to microgravity, deconditioning, and countermeasures. J. Physiol. Sci. 67, 271–281. doi: 10.1007/s12576-016-0514-8, PMID: 28000175 PMC10717636

[ref33] Tarantino U. Baldi J. Scimeca M. Piccirilli E. Piccioli A. Bonanno E.. (2016). The role of sarcopenia with and without fracture. Injury 47(Suppl. 4), S3–S10. doi: 10.1016/j.injury.2016.07.057 27496721

[ref34] Tarantino U. Cariati I. Marini M. D’Arcangelo G. Tancredi V. Primavera M.. (2020). Effects of simulated microgravity on muscle stem cells activity. Cell. Physiol. Biochem. 54, 736–747. doi: 10.33594/000000252, PMID: 32749090

[ref35] Tarantino U. Piccirilli E. Fantini M. Baldi J. Gasbarra E. Bei R. (2015a). Sarcopenia and fragility fractures: molecular and clinical evidence of the bone-muscle interaction. J. Bone Joint Surg. Am. 97, 429–437. doi: 10.2106/JBJS.N.00648, PMID: 25740034

[ref36] Tarantino U. Scimeca M. Piccirilli E. Tancredi V. Baldi J. Gasbarra E.. (2015b). Sarcopenia: a histological and immunohistochemical study on age-related muscle impairment. Aging Clin. Exp. Res. 27(Suppl. 1), 51–60. doi: 10.1007/s40520-015-0427-z 26197719

[ref37] Thomas M. Langley B. Berry C. Sharma M. Kirk S. Bass J.. (2000). Myostatin, a negative regulator of muscle growth, functions by inhibiting myoblast proliferation. J. Biol. Chem. 275, 40235–40243. doi: 10.1074/jbc.M004356200, PMID: 10976104

[ref38] van Loon J. J. W. A. (2007). Some history and use of the random positioning machine, RPM, in gravity related research. Adv. Sp. Res. 39, 1161–1165. doi: 10.1016/j.asr.2007.02.016

[ref39] Wang X. Pickrell A. M. Rossi S. G. Pinto M. Dillon L. M. Hida A.. (2013). Transient systemic mtDNA damage leads to muscle wasting by reducing the satellite cell pool. Hum. Mol. Genet. 22, 3976–3986. doi: 10.1093/hmg/ddt251, PMID: 23760083 PMC3766186

[ref40] Wehland M. Steinwerth P. Aleshcheva G. Sahana J. Hemmersbach R. Lützenberg R.. (2020). Tissue engineering of cartilage using a random positioning machine. Int. J. Mol. Sci. 21:9596. doi: 10.3390/ijms21249596, PMID: 33339388 PMC7765923

[ref41] Wuest S. L. Richard S. Kopp S. Grimm D. Egli M. (2015). Simulated microgravity: critical review on the use of random positioning machines for mammalian cell culture. Biomed. Res. Int. 2015:971474. doi: 10.1155/2015/971474, PMID: 25649075 PMC4310317

[ref42] Xu H. Ranjit R. Richardson A. Van Remmen H. (2021). Muscle mitochondrial catalase expression prevents neuromuscular junction disruption, atrophy, and weakness in a mouse model of accelerated sarcopenia. J. Cachexia. Sarcopenia Muscle 12, 1582–1596. doi: 10.1002/jcsm.12768, PMID: 34559475 PMC8718066

