# Supplementary material for: Role of Myostatin in Muscle Degeneration by Random Positioning Machine Exposure: An in vitro Study for the Treatment of Sarcopenia
Source: Front Physiol. 2022 Feb 3;13:782000. doi: 10.3389/fphys.2022.782000 (PMC8853288; doi:10.3389/fphys.2022.782000)
Supplement: Supplementary file 1 [file Image_1.PDF]

Supplementary Material

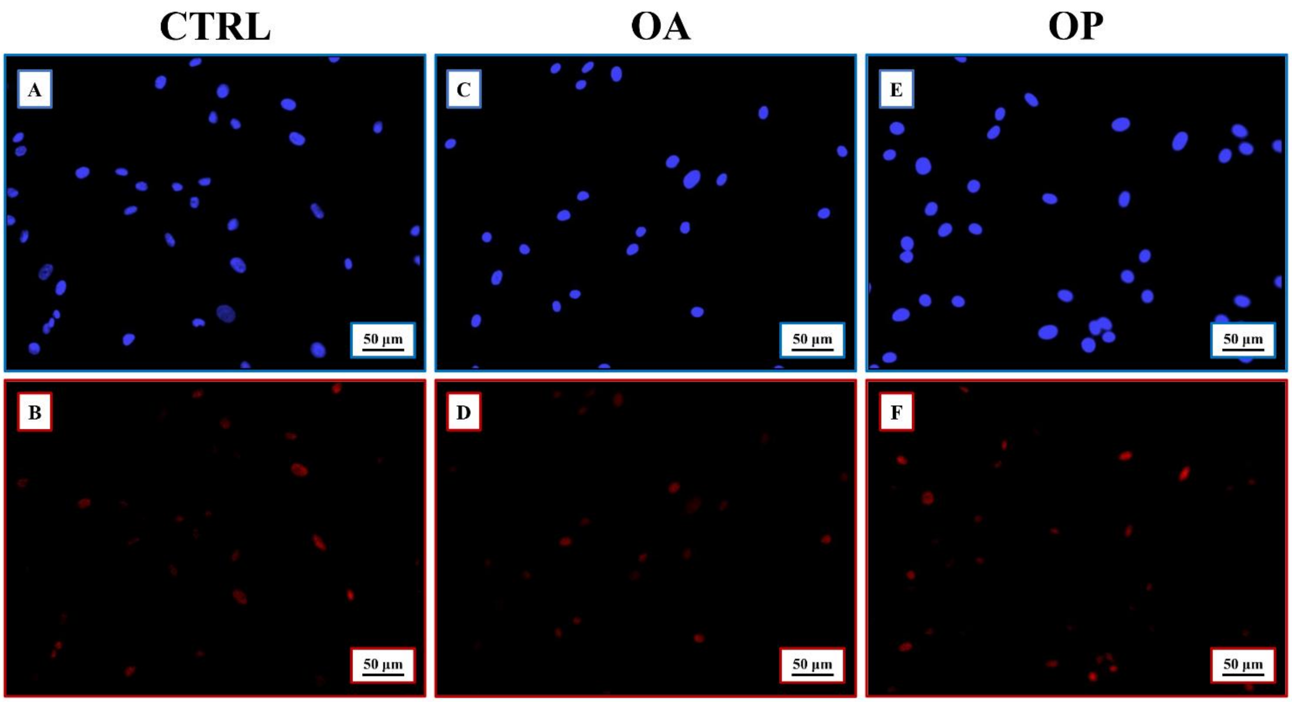

Supplementary Figure 1. Identification by immunofluorescence staining of satellite cells from CTRL (A,B), OA (C,D) and OP (E,F) patients. Nuclei are stained with DAPI (blue) (A,C,E). Pax7 immunostaining is depicted in red (B,D,F). Scale bar: 50 µm.
